# A Clinical Case of Multisystem Inflammatory Syndrome After SARS-CoV-2 Infection Associated with Group A β-Hemolytic Streptococcus Coinfection and Venous Thrombosis in a Child with Congenital Thrombophilia

**DOI:** 10.3390/cimb47050334

**Published:** 2025-05-07

**Authors:** Zdravka Stoyanova, Katya Temelkova, Margarita Ganeva, Teodor Vasilev, Anna Dasheva-Dimitrova, Desislava Kibarova-Hristova, Stefan Stefanov

**Affiliations:** 1 Pediatric Rheumatology Department, University Children’s Hospital “Ivan Mitev”, 11 “Akademik Ivan Evstratiev Geshov” Blvd., 1606 Sofia, Bulgaria; 2 Pediatrics Department, Faculty of Medicine, Medical University of Sofia, 15 “Akademik Ivan Evstratiev Geshov” Blvd., 1431 Sofia, Bulgaria

**Keywords:** case report, MIS-C, GAS coinfection, hyperinflammation, hemorrhagic rash, thrombosis, thrombophilia

## Abstract

Multisystem inflammatory syndrome in children (MIS-C) is a rare, delayed hyperinflammatory response, which occurs 2–6 weeks after SARS-CoV-2 infection. Main symptoms include fever, involvement of at least two organ systems, elevated inflammatory markers and evidence of infection with or exposure to SARS-CoV-2. While the prognosis is generally favorable, complications—such as myocardial dysfunction, coronary aneurysms, and coagulation disorders—can lead to severe outcomes, including death. Immunomodulatory and antithrombotic therapies are key components of treatment. We report a clinical case of a 3-year-old boy who developed MIS-C, initially presenting with fever, multiorgan involvement, and confirmed SARS-CoV-2 infection, along with a coinfection caused by group A β-hemolytic Streptococcus (GAS) isolated from throat culture. On the ninth day of illness, thrombosis of the right subclavian vein was detected. Subsequent genetic testing for thrombophilia revealed that the patient was a heterozygous carrier of *Factor V Leiden, Factor V HR2*, and *PAI-1 4G/5G polymorphisms*. Thromboembolic events (TEs) are serious and potentially life-threatening complications of MIS-C. This case highlights the occurrence of TE in a 3-year-old boy, an age group younger than typically observed, emphasizing the need for heightened awareness, early detection, and prompt intervention. Additionally, it underscores the importance of careful monitoring of thrombotic risks in MIS-C patients, particularly those with underlying prothrombotic conditions, to prevent severe outcomes.

## 1. Introduction

Multisystem inflammatory syndrome in children (MIS-C) is a severe, delayed hyperinflammatory response that occurs 2–6 weeks after SARS-CoV-2 infection [1]. It shares features of cytokine storm syndrome, characterized by elevated serum cytokine levels—primarily IL-6 and IL-10—along with neutrophilia, marked lymphopenia, and significant T-cell activation [2]. The diagnostic criteria for MIS-C include a fever lasting at least 24 h, accompanied by the involvement of at least two organ systems. These may include skin manifestations, lymphadenopathy, erythema or edema of the hands and feet, gastrointestinal symptoms, hypotension or shock, cardiac dysfunction, coagulopathy, elevated inflammatory markers, and a positive SARS-CoV-2 IgM/IgG or PCR test [1].

A study conducted between 2020 and 2022 on 31 Bulgarian patients with MIS-C [3] found that all cases presented with fever, with an average duration of five days. The most common clinical features included conjunctivitis and exanthema, each observed in 80% of patients. Gastrointestinal symptoms, such as vomiting and/or diarrhea, were reported in 70% of cases, while myocarditis was documented in 61%, and serositis in 51%. Additional symptoms included edema of the palms and feet (28%), periorbital edema (28%), cervical lymphadenitis (22%), and acute kidney injury (6%).

According to CDC data, between March 2020 and March 2023, 9370 cases of MIS-C were reported in the United States, with 76 fatalities [4]. Similarly, a multicenter study conducted across 22 countries between March 2020 and September 2022, which included 1009 patients, reported a mortality rate of 0.8%—identical to that observed in the U.S [5].

Venous thromboembolism (VTE) is a rare complication in children without underlying risk factors. The estimated annual incidence is between 0.07 and 0.14 per 10,000 healthy children, and 5.3 per 10,000 pediatric hospital admissions [6]. Along with hyperinflammation, MIS-C is marked by hypercoagulability and a higher risk of thrombotic events [7,8]. While reported incidence rates vary, approximately 7% of children with MIS-C are estimated to develop clinically significant thrombosis [9]. A literature review of 60 cases of MIS-C from 37 studies found that 91.7% of affected children had at least one risk factor for thrombosis. The most frequently observed risk factors were as follows: D-dimer > 5 times the upper limit of normal values (71.9%), pediatric intensive care unit hospitalization (61.7%), central venous catheter (36.7%), age > 12 years (36.7%), left ventricular ejection fraction < 35% (28.3%), mechanical ventilation (23.3%), obesity (23.3%), and extracorporeal membrane oxygenation (15%). Despite antithrombotic prophylaxis, 40% of MIS-C patients still develop TEs [10]. A concerning mortality rate of 28% has been reported in a small cohort of pediatric patients with COVID-19 or MIS-C who developed thromboses [11].

Intravenous immunoglobulin (IVIG), alone or in combination with corticosteroids (CS), is the first-line treatment for MIS-C. In cases with coronary involvement, IVIG helps reduce the risk of coronary artery lesions. The primary goal of treatment is to control the cytokine storm and inflammation. The best clinical outcomes are reported with IVIG at 1–2 g/kg/day (maximum dose: 100 g/day) and corticosteroids at 1–2 mg/kg/day [12,13]. Patients with platelet counts > 450 × 10⁹/L, coronary aneurysms, and/or thrombosis should receive antiplatelet and/or anticoagulant therapy [14].

Group A β-hemolytic Streptococcus (GAS) is a type of bacteria that causes various infections, from superficial infections, such as pharyngitis and impetigo, to invasive infections, such as sepsis, necrotizing myositis and fasciitis, scarlet fever and streptococcal toxic shock syndrome (STSS). GAS can also be associated with post-infectious inflammatory reaction (PIIR) [15]. As with most infectious diseases, the pathogenesis of invasive GAS is a complex interaction between the virulence factors of the pathogen and the host response.

We present a clinical case of a 3-year-old boy with MIS-C and a positive throat culture for GAS, complicated by thrombosis of the v. subclavia dextra.

## 2. Case Report

A 3-year-old boy initially presented with high-grade remittent fever. Laboratory markers showed leukocytosis (20 × 10^9^/L) and markedly elevated C-reactive protein (CRP, 320 mg/L). The boy was admitted three days after the onset of fever to a Pediatric Department. Further evaluation revealed acute kidney injury, hemostasis abnormalities, electrolyte imbalance, and a positive rapid antigen test for GAS. A chest X-ray showed a bilateral paracardial enhanced lung pattern. Treatment with ampicillin/sulbactam and corticosteroid was initiated. However, despite the treatment, the fever persisted, and the patient developed ecchymoses on the face, a petechial rash, and edema over the distal parts of the limbs.

**Figure 1:**
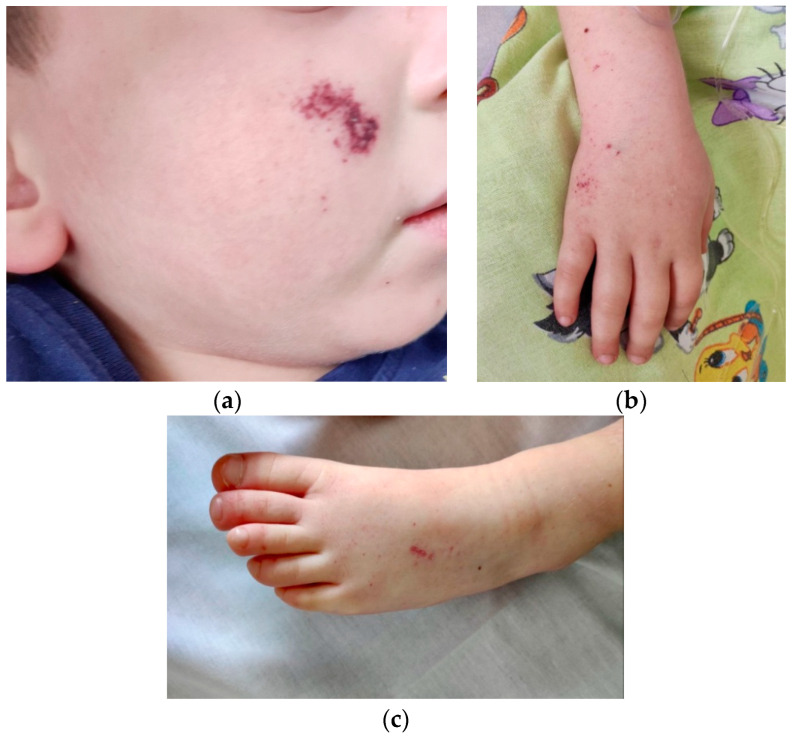
(**a**) Hemorrhagic lesion on right cheek. (**b**,**c**) Petechiae over upper and lower limbs.

On the fifth day of the disease, the child was transferred to the Intensive Care Unit (ICU) of the University Children’s Hospital “Ivan Mitev”. On admission he remained febrile (38 °C) and irritable, with a hemorrhagic lesion on the right cheek, petechiae on the upper and lower extremities, a generalized macular rash, swollen palms and feet, red and cracked lips, and erythema of the pharyngeal mucosa (Figure 1). Hepatosplenomegaly was also noted. No abnormalities of the respiratory, cardiovascular and nervous systems were detected.

The laboratory tests revealed leukocytosis with granulocytosis, lymphopenia, and thrombocytopenia with extremely elevated inflammatory markers—CRP 503.2 mg/L, ferritin 8494.2 ng/mL, hypoalbuminemia, and elevated liver enzymes, elevated D-dimer ≥ 5× upper limit of normal. Virological tests detected a positive result for SARS-CoV-2—IgM/IgA and IgG antibodies against the Nucleocapsid Protein (Table 1). At this early outpatient assessment, an extended antinuclear antibody (ANA) panel was also conducted and returned a negative result.

Microbiological cultures from the throat swab, urine, and blood culture did not identify any pathogenic agents. Abdominal ultrasound confirmed hepatosplenomegaly, while echocardiography showed no cardiac involvement.

**Figure 2:**
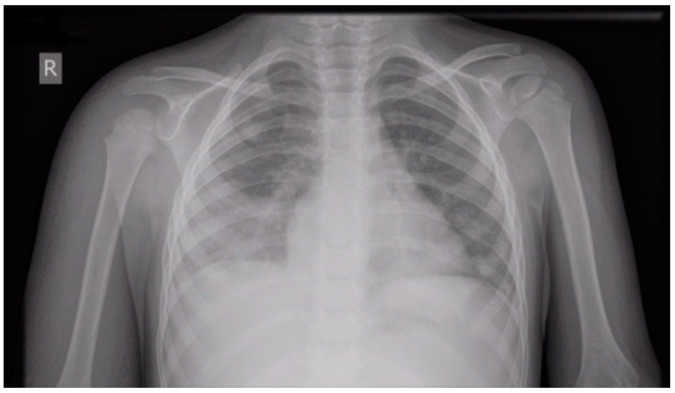
Right-sided pneumonia with pleural effusion.

**Figure 3:**
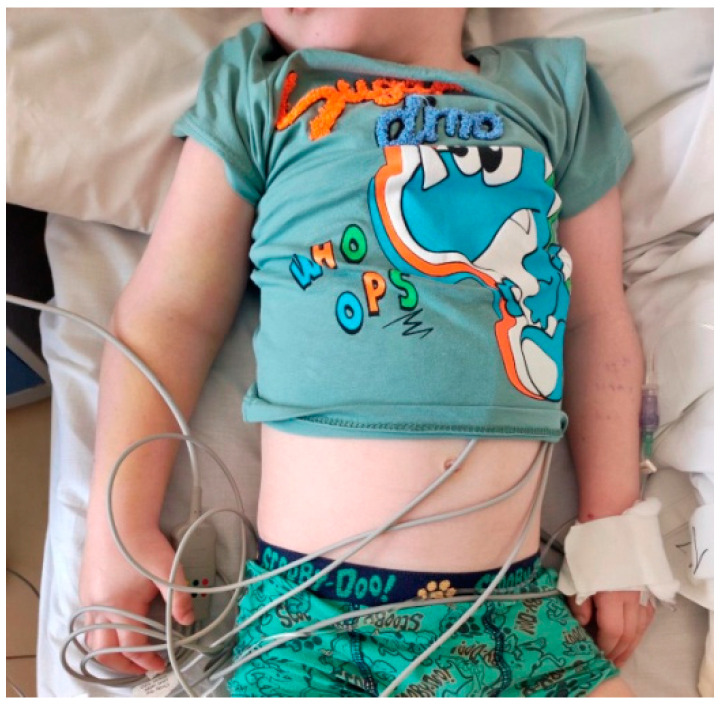
Edema of the right upper extremity.

On the first day of admission to the ICU, antibiotic therapy with ceftriaxone and vancomycin, along with methylprednisolone in a dosage of 2 mg/kg/day, was continued. On the third day, the child suddenly presented with changes in consciousness, along with dyspnea and tachypnea (54/min). Bronchial breath sounds were auscultated over the right lung base. Chest X-ray showed right-sided pneumonia with pleural effusion (Figure 2). The right hand was with hard swelling, warm skin, and hyperemia (Figure 3). The initial venous Doppler ultrasound evaluation of the upper extremity, performed by a vascular surgeon, showed no signs of thrombosis. Cardiac ultrasound revealed mitral insufficiency due to papillary muscle dysfunction and a borderline enlargement of the left ventricle, with normal myocardial contractility and normal pulmonary pressure. In response, spironolactone and captopril were added to the treatment plan.

**Figure 4:**
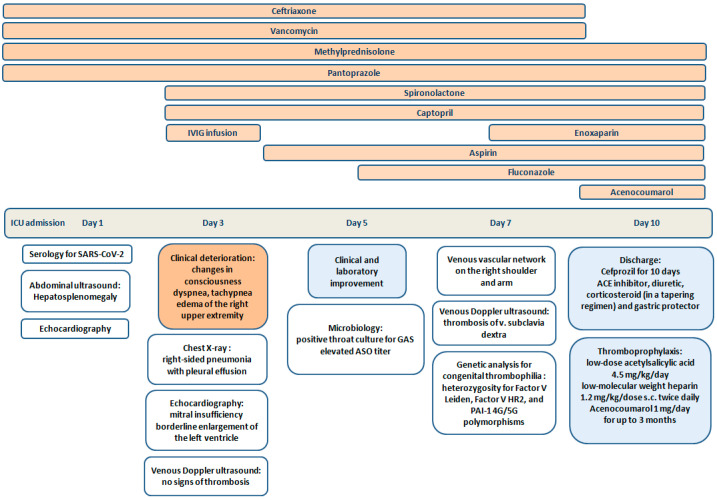
Therapeutic plan during the hospital stay.

Based on the child’s medical history as well as clinical and laboratory signs (fever, multiple organ system dysfunction including cardiovascular, mucocutaneous, neurologic, hematologic involvement and respiratory failure, increased inflammatory markers, and positive result for SARS-CoV-2 IgM/IgG), a diagnosis of MIS-C related to COVID-19 infection was made. According to the guidelines, IVIG infusion at a dose of 2 g/kg was administered (Figure 4).

**Figure 5:**
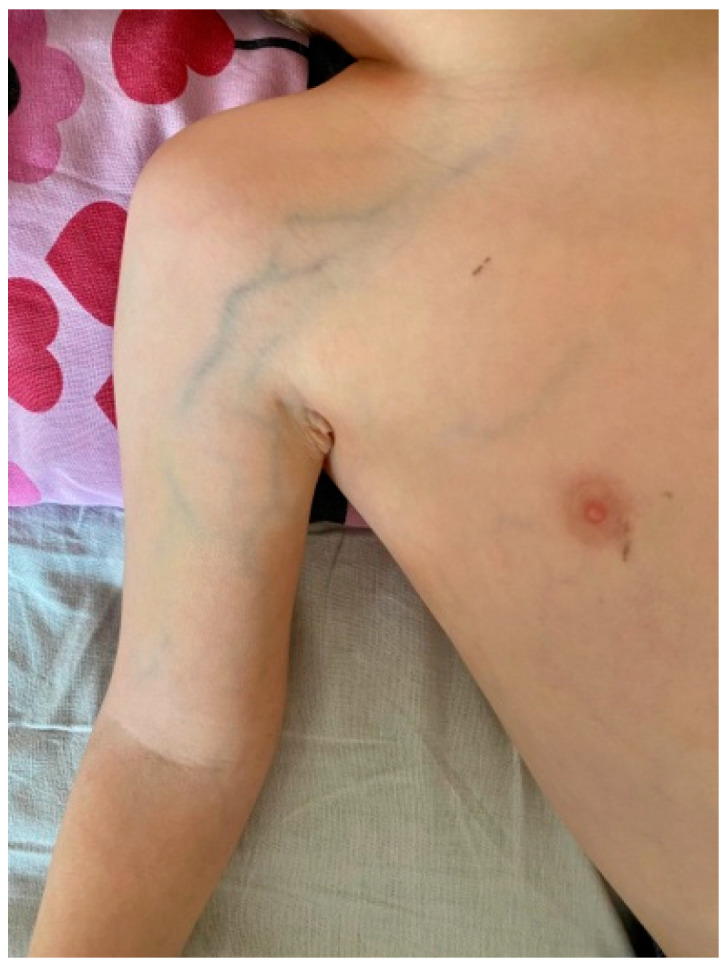
Dilated venous network.

On the 5th day of the hospitalization, the patient’s clinical condition as well as laboratory parameters remarkably improved. He was transferred to the pediatric rheumatology department with reduced swelling in the upper right limb, residual rash, and lamellar desquamation on the fingers and toes. A controlled throat culture was positive for GAS, with a slightly elevated antistreptolysin O (ASO) titer; however, it was extremely elevated compared to the initial value (rising from 6.16 to 178.52). During the hospital stay, a dilated venous vascular network was developed on the right shoulder and arm, prompting a new consultation with a vascular surgeon (Figure 5). Venous ultrasound confirmed thrombosis of v. subclavia dextra. An anticoagulant therapy was initiated with enoxaparin 1.2 mg/kg/dose s.c. twice daily and titrated to maintain an anti-Xa level of 0.5–1.0 anti-Xa U/mL.

Testing for thrombophilia revealed that the patient is a heterozygous carrier of several gene variants: *Factor V Leiden, PAI-1 4G/5G, MTHFR A1298C, MTHFR C677T, Factor V HR2 gene variants*. The family history for VTE was unremarkable.

The boy was discharged on the 10th day of the hospitalization in improved general condition, with no evidence of inflammatory activity and normal biochemical and coagulation parameters, along with improved pulmonary findings. At discharge, the therapy included an oral antibiotic, ACE inhibitors and diuretics, corticosteroid (in a tapering regimen), and a gastric protector. For outpatient thromboprophylaxis, the following were prescribed: low-dose acetylsalicylic acid (4.5 mg/kg/day), low-molecular weight heparin (LMWH) (1.2 mg/kg/dose s.c. twice daily), and Acenocoumarol (1 mg/day) for up to 3 months.

## 3. Discussion

MIS-C involves multiple organ systems and results from an overwhelming hyperinflammatory immune response. It shares several clinical and laboratory features with toxic shock syndrome. Similarly to Staphylococcus aureus and Streptococcus pyogenes, SARS-CoV-2 is believed to produce a superantigen (SAg), with the spike protein hypothesized to play a role in this process [16,17].

In the presented clinical case, the isolation of Group A Streptococcus and the elevated ASO titer indicate a recent streptococcal infection. Given the simultaneous presence of both a systemic GAS infection and a hyperinflammatory syndrome following prior SARS-CoV-2 infection, the overall clinical presentation is most accurately characterized as a coinfection.

The sudden deterioration in the patient’s clinical condition, along with chest radiology findings of right-sided pneumonia with pleural effusion, could suggest either streptococcal pneumonia or pulmonary vasculitis related to MIS-C, an extremely rare condition with only a few cases described in the literature [18]. Another possible condition that have been discussed was PIIR.

Post-infectious inflammatory reactions (PIIR) are characterized by the onset of clinical inflammatory manifestations occurring between 3 and 15 days after the resolution of the initial infectious episode. Clinical manifestations of PIIR include fever > 38 °C, arthritis, reactional pleural effusion, intraabdominal effusion, orchitis or epididymitis, and cutaneous manifestation (petechiae, erythema nodosum, vesicular eruptions, scarlatiniform eruptions). According to a study by Abraham et al. [15], PIIR was associated with pleuropneumonia, ICU hospitalization, and elevated CRP levels.

Notably, in the clustered cases reported by Spain’s multicenter network for invasive GAS analysis (Ped-GAS-net) in late 2022, there was a noticeable trend towards younger age groups and an increased frequency of pneumonia and pleural effusion. Additionally, there was a significant rise in ICU admissions for invasive GAS compared to the years preceding the pandemic [19,20].

We need to assess the potential of GAS and the SARS-CoV-2 spike protein as superantigens in amplifying the inflammatory response, contributing to more severe outcomes, and increasing the risk of complications. Further research is required to better understand the mechanisms of bacterial-viral coinfection and the role of these two superantigens.

Sepsis is one of the hyperinflammatory conditions that can mimic the manifestations of MIS-C. Both conditions present with fever, elevated inflammatory markers, hypotension/shock, coagulopathy, and multiorgan dysfunction [21]. It is important to note that MIS-C is a diagnosis of exclusion, requiring the ruling out of bacterial causes of inflammation, such as sepsis. For this reason, all patients are initially treated with antibiotics as if for sepsis.

In the presented clinical case, we observed laboratory evidence of a hyperinflammatory and prothrombotic state, including disseminated intravascular coagulation (DIC), which is associated with disease severity. DIC results from dysregulated immunothrombosis driven by systemic inflammation and excessive activation of coagulation pathways, similar to what is observed in multisystem inflammatory syndrome in children and sepsis.

The Streptococcus pyogenes M1 protein promotes the release of pro-coagulant microvesicles and, when complexed with fibrinogen, triggers NET formation, enhancing thrombosis. NETs facilitate platelet activation and aggregation, creating a procoagulant feedback loop [22,23,24]. Similarly, COVID-19 and MIS-C induce hypercoagulability and thromboembolic events, with complement activation implicated in thrombosis [25]. A study conducted in pediatric patients found that the rates of symptomatic venous thromboembolism (VTE) were 7% among patients aged 12–21 years and 1.3% among those aged 5–12 years [26]. Only a limited number of cases have been reported involving children under 5 years of age with MIS-C complicated by thrombosis, including occurrences of cerebral infarctions and intracardiac thrombi [27,28]. Patients with certain clinical factors (age ≥ 12 years, MIS-C) and laboratory markers (elevated D-dimers, especially ≥5× the upper limit of normal) have been identified as having independent risk factors for thrombosis. Thrombosis occurred in 6.5% of 138 patients with MIS-C, which is 13 times higher than the baseline risk [11]. The presence of additional risk factors, such as pediatric ICU admission, central venous catheter use, mechanical ventilation, and prolonged immobilization, can further promote hypercoagulability and elevate the risk of thrombosis, highlighting the importance of thromboprophylaxis [9,10]. Mortality rates are higher in children who develop thrombosis [9].

This case adds insight to the understanding of thromboembolic risk in MIS-C by documenting a rare occurrence in a 3-year-old—an age group younger than typically associated with TEs. The patient presented with three key risk factors for thrombosis: markedly elevated D-dimer levels (greater than five times the upper limit of normal), admission to the pediatric ICU, and an underlying congenital thrombophilia. Genetic analysis identified heterozygosity for *Factor V Leiden, Factor V HR2*, and *PAI-1 4G/5G polymorphisms*. While each of these prothrombotic mutations has been individually associated with increased thrombotic risk [29,30], their combined presence in a young child with thrombosis is exceptionally uncommon and, to our knowledge, has not been previously reported. This case highlights the potential for synergistic genetic risk in pediatric thrombosis and underscores the importance of considering comprehensive thrombophilia screening in children presenting with thrombotic events, particularly when additional clinical risk factors are present. Moreover, the presence of a concurrent GAS coinfection—an uncommon finding in the context of MIS-C, further underscores the complexity of this case. The deep vein thrombosis (DVT) treatment strategy in this case aligns with the American College of Rheumatology guidelines for multisystem inflammatory syndrome in children (MIS-C) associated with SARS-CoV-2, as well as the American Society of Hematology’s 2018 guidelines for the management of pediatric venous thromboembolism [31].

## 4. Conclusions

MIS-C is a diagnosis of exclusion, necessitating a comprehensive evaluation of differential diagnoses, as its management remains a challenge for physicians. In cases presenting with fever, organ involvement, and elevated inflammatory markers, empirical antibiotic therapy should be initiated to cover potential bacterial infections. Patients with MIS-C requiring ICU admission often exhibit clinical similarities to those with sepsis, a condition associated with higher incidence, morbidity, and mortality.

Thromboembolic events are severe, potentially life-threatening complications of MIS-C. Current pediatric thrombosis guidelines are often derived from adult data and typically focus on isolated risk factors. This case illustrates how the coexistence of multiple genetic mutations—*Factor V Leiden, Factor V HR2*, and *PAI-1 4G/5G polymorphisms*—may collectively increase thrombotic susceptibility in children. It underscores the importance of recognizing cumulative genetic risk and supports the consideration of multiplex thrombophilia testing in selected pediatric patients with VTE, particularly when additional clinical risk factors are present. Understanding how multiple inherited thrombophilic mutations interact in the pathogenesis of pediatric venous thromboembolism remains limited. This case contributes to that understanding and may aid in identifying children who could benefit from earlier or more intensive prophylaxis and screening.

Given the paucity of reported cases in children under 5 years of age, each additional case provides important contributions to the understanding of thromboembolic risk factors, clinical manifestations, and management strategies in young patients with MIS-C.

## Figures and Tables

**Table 1:** Laboratory evaluation of the patient.

	Normal Ranges	Day 1 *	Day 3	Day 5 **	Day 7
White blood count	5.5–15.5 × 10^9^/L	37.43	19.26	24.99	25.79
Lymphocytes	29–65%	3.4	17.1	15.5	17.7
Neutrophils	30–60%	94.5	76.4	82.1	79.9
Red blood count	3.9–5.1 × 10^12^/L	4.51	3.06	3.57	3.76
Hemoglobin	111–143 g/L	114	75	87	93
Platelets	286–509 × 10^9^/L	82	74	430	633
Erythrocyte sedimentation rate	1–18 mm/h	35	–	–	30
C-reactive protein	0–5 mg/L	503.27	>200	20.4	20.5
Procalcitonin	<0.1 ng/mL	42.29	–	14.58	0.3
Ferritin	4.4–64 ng/mL	8494.2	667	–	509
Uric acid	0–420 µmol/L	610	289	–	256
Albumin	38–54 g/L	31.99	–	–	35.77
Troponin I	0–1 ng/mL	<0.01	0.011	0.003	<0.01
NT-pro BNP	<300 pg/mL	9105	1357	420	302
ASAT	<50 U/L	159	–	–	15
ALAT	<36 U/L	64	–	–	12
LDH	120–300 U/L	975	284	–	324
Antistreptolysin O titer	0–150 IU/mL	6.16	–	–	178.52
Fibrinogen	200–400 mg/dL	354	274	–	314
D-dimer	<500 ng/mL	>3000	2878	1895	936.5
aPTT	28–34 s	47.4	24.3	24.7	25.9
INR	0.9–1.2	2.06	1.37	1.28	1.07
Prothrombin time	11–14 s	27	18.7	17	12.7
SARS-CoV-2 IgM/IgA	>0.6 BAU/mL	1.78	–	–	–
SARS-CoV-2 IgG	>1.6 BAU/mL	8.4	–	–	–

* at ICU admission. ** after IVIG infusion.

## Data Availability

The data presented in this study are available on request from the corresponding author.
